# Inequity in quality of life among culturally and linguistically diverse children in Australia

**DOI:** 10.1007/s11136-026-04309-6

**Published:** 2026-06-15

**Authors:** Alison Hayes, Li Ming Wen, Kirsten Howard, Louise Baur, Anagha Killedar

**Affiliations:** 1 Sydney School of Public Health, Faculty of Medicine and Health, University of Sydney, Sydney, NSW 2006 Australia; 2 Health Promotion Unit, Population Health Research & Evaluation Hub, Sydney Local Health District, Sydney, NSW 2037 Australia; 3 Leeder Centre for Health Policy Economics and Data, Faculty of Medicine and Health, University of Sydney, Sydney, NSW 2006 Australia; 4 Sydney Medical School, University of Sydney, Sydney, NSW 2002 Australia

**Keywords:** Quality of life, Cultural diversity, Children, Australia

## Abstract

**Purpose:**

The aims of this study were to investigate whether children from different culturally and linguistically diverse (CALD) backgrounds in Australia have similar Health Related Quality of Life (HRQoL), in early childhood, middle childhood, and adolescence.

**Methods:**

We used data from 9099 children from the Longitudinal Study of Australian Children (LSAC), aged between 2 and 17 years, including HRQoL measured with the parent proxy Pediatric Quality of Life Inventory (PedsQL). The study pertained to cohort data from 2004 to 2018. CALD groups were defined according to child, mother’s and father’s country of birth and main language spoken at home. The association of child HRQoL with CALD group, was analysed using generalised estimating equations, adjusted for age, sex, socioeconomic position (SEP) and weight status.

**Results:**

Children of Middle Eastern or North African, South-East Asian, South and Central Asian and Oceania backgrounds had significantly lower HRQoL (*p* < 0.05) than children from English speaking backgrounds. These disparities were greatest during middle childhood and adolescence and only partly explained by lower SEP and weight status. Disparities in physical HRQoL were greater than psychosocial HRQOL.

**Conclusion:**

Considerable inequity in HRQoL is present in Australian children from different CALD backgrounds. This study highlights the need for culturally tailored programs for school-aged children to improve their physical HRQoL.

**Supplementary Information:**

The online version contains supplementary material available at https://doi.org/10.1007/s11136-026-04309-6.

## Abstract (plain language summary)

Health-related quality of life is an important measure of child overall health and includes physical and psychosocial health. There is evidence for lower quality of life in some culturally diverse populations, but no studies have examined this in children and adolescents. We used data from a very large Australian study that measured quality of life of children from age 2 to age 17 years and found lower quality of life among some cultural groups that developed primarily in middle childhood. Disparities in physical HRQoL were greater than psychosocial HRQOL. This highlights the need for culturally tailored programs for school-aged children to improve their physical HRQoL.

**Supplementary Information:**

The online version contains supplementary material available at https://doi.org/10.1007/s11136-026-04309-6.

## Introduction

Australia is a country with a high level of cultural and linguistic diversity and ongoing high immigration patterns. Children from culturally and linguistically diverse (CALD) households and those living with socio-economic disadvantage are defined as priority populations in Australia based on increased risk of health inequity, arising from the social determinants of health [1]. The evidence for child health inequities based on socio-economic position (SEP) is well established both in Australia and globally [2, 3]. There is less evidence regarding inequities in broader measures of child health, such as health-related quality of life (HRQoL), and few studies have examined these inequities among children from priority groups other than those living with socioeconomic disadvantage.

Internationally there is some evidence that children of migrants have a lower HRQoL than those of non-migrants. For example, Puder et al. [4] found that young children (2–6 years) of migrant parents living in Switzerland had significantly lower HRQoL than those with non-migrant parents. Similarly, in Greece, school-aged children of immigrant parents were found to have lower HRQoL [5]. In contrast, a study in Germany of pre-school children found no difference in HRQoL among migrant compared with native born children [6] and a study in Norway found children of immigrant families to have higher HRQoL [7]. Racial and ethnic disparities in HRQoL have also been found among children in the US [8, 9]. These studies have been conducted in either preschool-aged children or school-aged children, with no single study analyzing HRQoL throughout the child and adolescent life course. In Australia, lower SEP is associated with lower HRQoL in childhood [10, 11], but HRQoL has not been examined among Australian children from a diverse range of cultural and language groups.

Recent research has shown that children from some priority populations in Australia are at increased risk of being above a healthy weight [12], including those from Middle Eastern/North African (MENA), Aboriginal and Pacific households. There is also substantial evidence that overweight and obesity impact adversely on HRQoL during childhood [10, 13, 14] suggesting that weight status could contribute to inequalities in HRQoL between different population groups.

The aims of this study were to investigate whether children from different cultural and linguistic groups in Australia have similar HRQoL, and whether any differences are more apparent at any particular age. Given their established associations with HRQoL, we also aimed to determine the role of SEP and weight status in explaining any identified differences in HRQoL between CALD groups.

## Methods

### Participants

We conducted longitudinal analysis using data from the Longitudinal Study of Australian Children (LSAC) [15]. Participants were 9099 children and their parents including 1998 from CALD backgrounds and 7101 from English language backgrounds. The LSAC is the largest ongoing population study in Australia, following health and development of children. It began in 2004 and followed two cohorts of children: the ‘B’ (baby) cohort aged 0/1 years in wave 1 (2004) and the ‘K’ (kindergarten) cohort aged 4/5 years in wave 1. Children and caregivers were interviewed every 2 years. In the current analysis, we used 7 waves of data from the ‘B’ cohort, encompassing child age 2/3 years until 14/15 years, and 7 waves of data from the ‘K’ (kindergarten) cohort, covering child age 4/5 years to 16/17 years. Each child had between 1 and 7 (mean 5.3) observations of HRQoL, between 2004 and 2018.

### Measurement of health related quality of life

The outcome of interest was HRQoL measured with the Pediatric Quality of Life Inventory v4.0 Generic Core Scales (PedsQL) [16], one of the most widely used pediatric HRQoL instruments which has very good psychometric properties for the measurement of many chronic diseases in children [17], including in the context of overweight and obesity [18]. It consists of a 23-item scale which asks questions on physical (8 items) emotional (5 items), social (5 items) and school functioning (5 items). The Toddler version has just 21 items, as only 3 of the school functioning questions are included for this age group. Each item is scored on a 5-point scale (0 = never a problem; 1 = almost never a problem; 2 = sometimes a problem; 3 = often a problem; 4 = almost always a problem). Scores are reverse transformed to provide a Total Score from 0 to 100, with higher scores representing better HRQoL. A Psychosocial Health Summary Score may be calculated from the emotional, social and school functioning items and the Physical Health Summary Score is the same as the physical functioning score [16]. The Total, Psychosocial and Physical summary measures have good internal consistency reliability of 0.9, 0.88 and 0.86, respectively [16]. The PedsQL is available in both parent proxy and child report versions, and with age-appropriate versions [16], making it one of the few HRQol measures that can be used throughout the child and adolescent life course. Within the LSAC, 3 different age-appropriate versions of the PedsQL parent proxy report were administered.

### Demographic/cultural and language groups

CALD groups were defined according to country of birth of the child, mother and father and the main language spoken at home, guided by the Australian Bureau of Statistics (ABS) Standards for Statistics on Cultural and Language Diversity [19]. Nine country and language groups are defined in the ABS Standard Classification of Countries [20] and languages [21]. These are: (1) English-speaking countries; (2) Middle East and North Africa; (3) East and South-East Asia; (4) South and Central Asia; (5) Europe; (6) Sub-Saharan Africa; (7) Americas; (8) Oceania excluding Australia and New Zealand; and (9) Aboriginal and Torres Strait Islander peoples. Further information on the classification of these groups is provided in Lung et al. [12] and in Supplementary Table 1. The referent group in our analyses was the largest group, those from English speaking backgrounds, and CALD groups were defined by groups 2–8, above. Aboriginal and Torres Strait Islander children were not included in the present analysis.

### Socioeconomic position

SEP is defined within the LSAC [22] as a composite variable including household income, parents’ occupation and education status, provided as a z-score, from which we generated quintiles of SEP for our analyses.

### Weight status

In the LSAC, childrens’ heights and weights were measured at each visit by a trained research assistant using stadiometers and scales [23]. BMI-z was determined using WHO growth standards [24] and weight status groups defined using the following cut-points (underweight: BMI-z ≤ -1; healthy weight BMI-z > -1 and ≤ 1; overweight BMI-z > 1 and ≤ 2; obesity BMI-z > 2 ). As the proportion of children in the LSAC in an underweight category was very small (< 1%), they were included in the healthy weight category.

### Statistical methods

We investigated the association of the total PedsQL score with child CALD group, using data from the combined B and K cohorts. We used generalised estimating equations (GEE) to account for the repeated measures of HRQoL among the same children. The GEE models included robust variance estimation. Significance levels of *p* < 0.05 were used for main effects and *p* < 0.01 for interaction effects [25].

In the first analysis, we investigated the association of total PedsQL score with child CALD group after controlling for age and sex (Model 1). To assess whether SEP and weight status explained the associations observed in Model 1, we then further adjusted for SEP quintile and weight status (healthy, overweight or obesity) as confounders and examined any change in the coefficients for CALD groups (Model 2). We tested for interactions between CALD group and other significant covariates to identify any modifying effects. Using model 2, marginal predictions were used to predict adjusted PedsQL scores by cultural and demographic groups.

Finally, we investigated the association of the component parts of HRQoL with CALD group and whether any associations differed by child developmental age. GEE models were fitted for the association of the Physical Health Summary Score and the Psychosocial Health Summary Score with child CALD group (adjusted for sex, SEP quintile and weight status). We stratified all analyses by three periods of childhood, representing key development periods [26]: under 6 years (pre-school/early childhood), 6–11 years (middle childhood) and 12–17 years (adolescence).

## Results

Summary statistics of the analysis dataset by demographic group are shown in Table 1 and missing data by cohort and wave are shown in Supplementary Table 2. The analysis included 48,368 records of HRQoL, collected at repeated timepoints from 9099 children over a 15-year period. Mean PedsQL Total score was highest overall for children from English and European households and lowest for Middle Eastern/North African (MENA) and East Asian households. Children from MENA households had higher representation in the lowest SEP quintile than the other groups. The distribution of weight status groups was heterogeneous, with MENA, the Americas and Oceania groups having the lowest proportion of children in healthy weight.

**Table 1:** Summary statistics of child characteristics by CALD group, including all records over all waves, B and K cohorts

*N*	English	Middle East & North Africa	East Asia	South & Central Asia	Europe	Africa	Americas	Oceania	Total
	7101	251	553	211	644	87	97	155	9099
Total records	38,863	966	2617	1050	3355	380	488	649	48,368
PedsQL total scale score, mean (se)	80.6 (0.1)	76.0 (0.5)	76.1 (0.3)	77.6 (0.5)	80.1 (0.2)	79.1 (0.7)	79.7 (0.6)	78.0 (0.6)	80.1 (0.1)
Sex									
Male	51.2	51.6	50.7	57.4	52.1	32.4	50.8	61.0	51.3
Female	48.8	48.4	49.3	42.6	47.9	67.6	49.2	39.0	48.7
Age group (%)									
Early childhood	25.0	30.3	25.3	24.5	25.0	25.5	24.6	29.3	25.2
Middle childhood	45.1	46.0	44.4	44.1	45.6	45.5	45.2	45.2	45.1
Adolescence	29.9	23.6	30.3	31.5	27.4	29.0	30.2	25.5	29.7
SEP quintile (%)									
1 (lowest)	18.2	34.6	24.6	8.3	14.0	11.6	11.5	25.1	18.3
2	20.6	20.2	13.3	12.1	16.8	13.2	17.4	17.1	19.6
3	20.6	16.7	15.8	19.2	20.5	18.2	22.3	23.7	20.3
4	20.6	11.5	18.5	27.4	24.8	28.7	21.9	17.1	20.8
5 (highest)	20.0	17.0	27.7	33.0	23.9	28.4	26.8	16.9	21.0
Weight status (%)									
Healthy	68.2	53.6	72.3	74.5	66.0	72.1	55.7	52.8	67.8
Overweight	22.1	28.1	18.0	16.6	23.0	21.3	27.9	25.6	22.1
Obesity	9.7	18.2	9.7	8.9	11.0	6.6	16.4	21.6	10.1

Our GEE analysis (Model 1) indicated that children from MENA, East Asian, South and Central Asian, and Oceanian families had significantly lower HRQoL during childhood, than those from the referent population (Table 2). Girls had lower HRQoL than boys and HRQoL declined overall with increasing age (Table 2). After adjustment for SEP quintile and weight status (Model 2), these effects persisted, indicating SEP and weight status were not major drivers of the differences in HRQoL across CALD groups. SEP and weight status were independent predictors of HRQoL and, based on the coefficients in Model 2, a child with obesity, from a MENA household in the lowest SEP quintile, would be predicted to have a PedsQl total score 10 points lower than a child with healthy weight from an English speaking high SEP quintile household. The adjusted mean PedsQL scores for children from MENA, East Asian, South and Central Asian households (Model 2) were 76.4, 76.4, and 76.6 respectively, compared to 80.3 for the English referent group. Whilst these were statistically significant differences, they were slightly lower than the minimal clinically important difference ( MCID) of 4.5 points on the Total scale [17]. Interaction effects between CALD group and other demographic variables were not statistically significant indicating no evidence that the association of HRQoL and CALD group was modified by sex, weight status or SEP.

**Table 2:** GEE models for the association of PedsQl score and Cultural and linguistic groups

Characteristic	Model 1 ^a^			Model 2 ^b^		
	β Coefficient	se	*p*	β Coefficient	se	*p*
Age (years)	− 0.058	0.014	< 0.001	− 0.044	0.014	0.002
Female	− 0.483	0.215	0.025	− 0.608	0.212	0.004
CALD group						
English	referent	–	–	Referent	–	–
Middle East & North African	− 4.742	0.814	< 0.001	− 3.954	0.796	< 0.001
East Asian	− 4.061	0.514	< 0.001	− 3.995	0.514	< 0.001
South & Central Asian	− 3.362	0.863	< 0.001	− 3.754	0.857	< 0.001
European	− 0.247	0.412	0.548	− 0.321	0.408	0.431
African	− 1.085	1.230	0.378	− 1.302	1.235	0.292
Americas	− 0.377	1.103	0.733	− 0.227	1.105	0.837
Oceania	− 3.687	1.095	0.001	− 2.820	1.076	0.009
SEP quintile						
1 (most disadvantaged)	–	–	–	− 1.890	0.256	< 0.001
2	–	–	–	− 0.933	0.229	< 0.001
3	–	–	–	− 0.207	0.215	0.338
4	–	–	–	− 0.124	0.188	0.51
5	–	–	–	referent	–	–
Weight status						
Healthy	–	–	–	referent	–	–
Overweight	–	–	–	− 0.919	0.146	< 0.001
Obesity	–	–	–	− 4.029	0.269	< 0.001

^a^ Model 1 adjusted for age and sex; ^b^ Model 2 adjusted for age, sex, SEP quintile and weight status

**Fig. 1:**
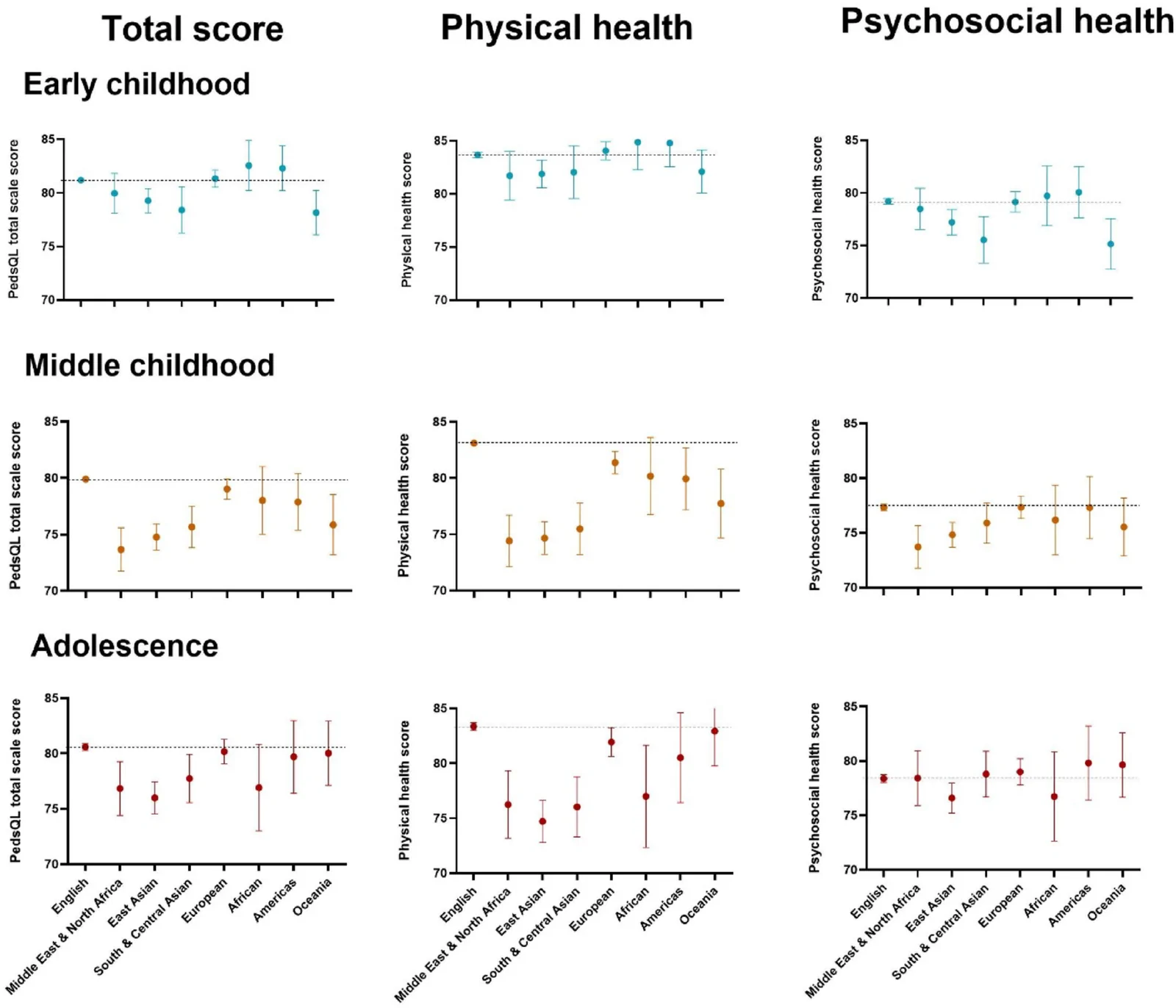
Marginal predictions of PedsQl Total scale score, Physical Health summary score and Psychosocial Health summary score by CALD group, stratified by age group. Dashed lines represent quality of life of the referent group

Examination of the three developmental periods of childhood indicated that inequalities in overall HRQoL among CALD populations were greatest during middle childhood and adolescence (Fig. 1 and Supplementary Table 3). Differences in Total Scale score compared with the referent group exceeded the MCID of 4.5 points for children over 6 years from MENA and East Asian backgrounds. There were greater disparities in physical health than psychosocial health in middle childhood and adolescence (Fig. 1 and Supplementary Tables 4 and 5), suggesting that the differences in overall HRQoL are mostly driven by disparities in physical health. For example, In middle childhood and adolescence, children from three CALD groups (MENA, East Asian and South Asian) had significantly lower Physical Health scores which also exceeded the MCID of 6.9 points [17]. Three CALD groups had significantly lower Psychosocial Health than the referent group (*p* < 0.05) but none of these differences reached the MCID of 5.49 [17].

## Discussion

We found considerable inequality in child HRQoL in Australia, with children of four CALD groups having significantly lower overall HRQoL in middle childhood and adolescence than children from English speaking backgrounds. Of these groups, children from MENA and South-East Asian households had disparities in HRQoL that exceeded the MCID. We consider the differences in HRQoL across CALD groups as unfair, i.e. they are inequities, as they are likely a result of sociocultural circumstances.

We found SEP and weight status to be independent predictors of HRQoL, meaning children from CALD groups associated with lower HRQoL, who are also from a low SEP quintile household would experience ‘double disadvantage’ in their lower HRQoL, which could be further exacerbated by weight status. The implication is that although culturally tailored obesity prevention in CALD communities may improve child HRQoL, and that improved socio-economic circumstances will also likely improve children’s HRQoL, neither on their own would be sufficient to equalise child HRQoL across cultural groups.

Few studies have examined HRQoL in relation to both SEP and cultural diversity. Our findings of disparities in HRQoL persisting even after controlling for SEP and weight status contrast with those of Wallander et al. who found that SEP completely explained the differences in HRQoL between African American and White children in the United States but not Hispanic children [27]. Pantzer et al. [28] found that the poorer HRQoL among adolescents of migrant parents in Spain, was mediated by socioeconomic disadvantage, whilst a study of children of migrants in Switzerland [4] found lower HRQoL to be only partially mediated by SEP. These differences may be attributed to the specific cultural and ethnic groups in the different studies, the different country contexts, and whether migrant groups were considered as one homogeneous group. In Australia, our results suggest that being a child of migrant parents is not, in itself, the pertinent factor, as we have found quite heterogeneous HRQoL among children of migrants from different cultural backgrounds. For example, children from a non-English speaking but European background did not have significantly different HRQoL to the English referent group (adjusted PedsQL Total score predictions 80.1 cf. 80.4) whilst children from MENA, South-East Asian, South and Central Asian CALD households had lower HRQoL scores (76.4, 76.4, 76.6 respectively). However, our findings are particular to the cultural groups in Australia and would not necessarily be generalisable to children in other country contexts.

We found that culturally-based disparities in HRQoL were more pronounced in middle childhood and adolescence than in early childhood, and perhaps surprisingly, disparities in physical HRQoL were greater than disparities in psychosocial HRQoL. The lower physical HRQoL of some CALD groups may be due to differences in health literacy [29] or cultural barriers to seeking healthcare for childhood chronic disease [30] that have flow on effects on physical health. Another possibility is that there are differences in access to appropriate physical health opportunities for children from some CALD groups [31]. The emergence of these physical HRQoL disparities in middle childhood and adolescence may be due to the rapid physical development at this time and increased peer comparison within school contexts. This suggests that schools have an important role in delivering physical education in a culturally acceptable way, with acknowledgment of social and cultural context, values, religious beliefs and traditions [32]. It also indicates the need for culturally tailored and co-designed physical and health education in schools.

### Strengths and weaknesses

This study is among the few internationally to examine inequity in child HRQoL for CALD population groups and the first study in Australia to examine these inequities over the entire child life course. There are many strengths of this study. It is based on a very large longitudinal cohort, with 9099 children, approximately 48,000 data points and repeated measures of HRQoL among the same children, which allows for rigorous statistical analysis. The PedsQL quality of life instrument used in this study is one of the most widely used pediatric quality of life instruments which has very good psychometric properties in the context of obesity and several common childhood conditions [33]. Additionally, the use of age-appropriate versions of PedsQL in LSAC, which have only small differences in wording, meant that HRQoL was measured in a consistent way and comparisons could be made over the entire childhood and adolescent life course. Measures of SEP used in the study were at the individual level, rather than area-based measures, and were measured at each wave of data collection. Similarly, height and weight were objectively measured.

However, it should be noted that the PedsQL was not developed in multi-ethnic/culturally diverse populations, and hence some of the domains may be less relevant to particular demographic groups, or pertinent culturally relevant domains of health and wellbeing may be missing. The English language version of the PedsQL was administered in the LSAC and the presence of interpreters was limited; hence there may have been bias in recruitment of linguistically diverse parents. It also should be noted that HRQoL measurement was by parent proxy report, and it is possible that parents’ estimation of children’s HRQoL may be different to that reported by children themselves. Indeed, some studies have found poor correlation between parent proxy and child reported HRQoL [9, 34], hence it is possible different results might be obtained with the child self-report version. Finally, the number of children in some demographic groups was small, so non-significant outcomes in some groups may have been due to insufficient power.

## Conclusion

We have shown that considerable inequity in HRQoL is present in Australian children, across cultural and linguistic groups, in particular during middle childhood and adolescence, and for domains of physical HRQoL. The study highlights the need for culturally tailored and co-designed physical and health education in schools.

## Supplementary Information

Below is the link to the electronic supplementary material.

Supplementary Material 1

Supplementary Material 2

## Data Availability

Data used in this study are available from the Longitudinal Studies Dataverse website ( [https://dataverse.ada.edu.au/dataverse/lsac](https:/dataverse.ada.edu.au/dataverse/lsac) ) for those who meet the criteria for access to de-identified LSAC data.
