# Supplementary material for: Inequity in quality of life among culturally and linguistically diverse children in Australia
Source: Qual Life Res. 2026 Jun 15;35(8):202. doi: 10.1007/s11136-026-04309-6 (PMC13269146; doi:10.1007/s11136-026-04309-6)
Supplement: Supplementary file 1 — Supplementary Material 1 [file 11136_2026_4309_MOESM1_ESM.docx]

**Supplementary Table 1 Classification of child cultural and language groups based on language spoken at home and country of birth for child and parents**

| Population | Country list | Language list |
| --- | --- | --- |
| English | Australia, United Kingdom, England, Scotland, Wales, Northern Ireland,  Ireland, New Zealand, Canada, USA, South Africa | English, Auslan |
| Middle East & North Africa | Algeria, Egypt, Libya, Morocco, Sudan, Bahrain, Gaza Strip and West Bank, Iran, Iraq, Israel, Jordan, Kuwait, Lebanon, Qatar, Saudi Arabia,  Syria, Turkey, United Arab Emirates | Kurdish, Dari, Iranic not elsewhere classified, Arabic, Assyrian including Aramaic, Hebrew, Middle Eastern Semitic Languages not  elsewhere classified, Turkish |
| East Asia | Myanmar, Cambodia, Laos, Thailand, Vietnam, Brunei Darussalam, Indonesia, Malaysia, Philippines, Singapore, Timor-Leste, China (excludes SARs and Taiwan), Hong Kong (SAR of China), Macau (SAR of China), Taiwan, Japan, South Korea | Burmese, Burmese and related languages not elsewhere classified, Hmong-Miean, Khmer, Vietnamese, Mon-Khmer, Lao, Thai, Cebuano, Ilokano, Indonesian, Malay, Tetum, Tagalog, Other Southeast Asian languages, Cantonese, Hakka, Hokkien, Teochew, Mandarin, Chinese  not elsewhere classified, Japanese, Korean, |
| South and Central Asia | Bangladesh, India, Maldives, Nepal, Pakistan, Sri Lanka, Afghanistan, Armenia, Azerbaijan, Georgia, Kazakhstan, Kyrgyzstan, Tajikistan,  Turkmenistan, Uzbekistan | Kannada, Malayalam, Tamil, Telegu, Bengali, Gujurati, Hindi, Konkani, Marathi, Nepali, Punjabi, Sindhi, Sinhalese, Urdu, Indo-Aryan not  elsewhere classified, Other Southern Asian langauges |
| Europe | Austria, Belgium, France, Germany, Luxembourg, Netherlands, Switzerland, Denmark, Finland, Norway, Sweden, Gibraltar, Italy, Malta, Portugal, Spain, Albania, Bosnia and Herzegovina, Croatia, Cyprus, North Macedonia, Greece, Moldova, Romania, Slovenia, Serbia, Czechia, Estonia, Hungary, Lithuania, Poland, Russia Federation, Slovakia,  Ukraine | Danish, Norwegian, Swedish, Finnish, French, Greek, Portuguese, Spanish, Italian, Maltese, Latvian, Hungarian, Lithuanian, Russian, Ukrainian, Bosnian, Croatian, Slovene, Serbian, Polish, Slovak, Romanian, Albanian, Other Eastern European languages not elsewhere classified |
| Africa | Ghana, Liberia, Nigeria, Sierra Leone, Angola, Eritrea, Ethiopia, Kenya, Malawi, Mauritius, Namibia, Seychelles, Somalia, Eswatini, Tanzania, Uganda, Zambia, Zimbabwe, Southern and Eastern Africa, not  elsewhere classified | Acholi, Mauritian Creole, Oromo, Somali, Swahili, Amharic, Tigre, African languages not elsewhere classified, French |
| Americas | South America, no further description, Argentina, Bolivia, Brazil, Chile, Colombia, Ecuador, Guyana, Peru, Uruguay, Venezuela, Costa Rica, El Salvador, Honduras, Mexico, Nicaragua, Bahamas, Caribbean, no further  description, Jamaica, Trinidad and Tobago | Spanish, Portuguese, English |
| Oceania | New Caledonia, Papua New Guinea, Solomon Islands, Melanesia/Polynesia not further described, Cook Islands, Fiji, Niue,  Samoa, Samoa, American, Tonga | Fijian, Maori Cook Island, Maori New Zealand, Motu, Niue, Samoan, Tongan, Pacific Austronesian languages not elsewhere classified,  Oceanian Pidgins and Creoles not elsewhere classified Kiwai |

**Supplementary Table 2. Numbers of participants, missing data and total analysis population for analysis of health-related quality of life data**

|  | **Early childhood** | | **Middle Childhood** | | | **Adolescence** | | |
| --- | --- | --- | --- | --- | --- | --- | --- | --- |
| **Age (years)** | **2/3** | **4/5** | **6/7** | **8/9** | **10/11** | **12/13** | **14/15** | **16/17** |
| **K Cohort Wave** |  | 1 | 2 | 3 | 4 | 5 | 6 | 7 |
| **Total participants** |  | 4753 | 4273 | 4168 | 3913 | 3101 | 3203 | 2799 |
| **Missing PedsQL** |  | 714 | 905 | 486 | 32 | 53 | 76 | 73 |
| **Missing BMI** |  | 17 | 8 | 1 | 14 | 1 | 5 | 0 |
| **Missing SEP** |  | 9 | 18 | 13 | 31 | 37 | 31 | 69 |
| **Missing CALD group** |  | 0 | 0 | 0 | 0 | 0 | 0 | 0 |
| **Analysed K cohort** |  | 4021 | 3353 | 3671 | 3844 | 3,619 | 3105 | 2688 |
|  |  |  |  |  |  |  |  |  |
| **B Cohort Wave** | 2 | 3 | 4 | 5 | 6 | 7 | 8 |  |
| **Total participants** | 4346 | 4178 | 4037 | 3865 | 3476 | 3090 | 2859 |  |
| **Missing PedsQL** | 960 | 506 | 19 | 46 | 37 | 32 | 32 |  |
| **Missing BMI** | 15 | 12 | 11 | 9 | 8 | 0 | 1 |  |
| **Missing SEP** | 15 | 17 | 19 | 39 | 28 | 17 | 10 |  |
| **Missing CALD group** | 0 | 0 | 0 | 0 | 0 | 0 | 0 |  |
| **Analysed B cohort** | 3367 | 3653 | 3988 | 3779 | 3411 | 3048 | 2821 |  |
| **TOTAL Analysis Population** | **3367** | **7674** | **7341** | **7450** | **7255** | **6667** | **5926** | **2688** |

**Supplementary Table 3 GEE models for the association between** **PedsQl Total score and Cultural and linguistic group, stratified by child age, and with adjustment for sex, SEP and weight status.**

| **Characteristic** | **Under 6**  **N=9099** | | | **6-11 years**  **N=8310** | | | **12-17 years**  **N=6791** | | |
| --- | --- | --- | --- | --- | --- | --- | --- | --- | --- |
|  | **β Coefficient** | **se** | **p** | **β Coefficient** | **se** | **p** | **β Coefficient** | **se** | **p** |
| **Female** | 0.437 | 0.222 | 0.049 | -0.0901 | 0.247 | 0.713 | -2.194 | 0.301 | **<0.001** |
| **CALD group** |  |  |  |  |  |  |  |  |  |
| English | referent | - | - | referent | - | - | referent | - | - |
| Middle East & North African | -1.236 | 0.954 | 0.195 | -5.876 | 0.978 | **<0.001** | -3.149 | 1.248 | **0.012** |
| East Asian | -1.924 | 0.594 | **0.001** | -5.144 | 0.609 | **<0.001** | -4.789 | 0.757 | **<0.001** |
| South & Central Asian | -2.788 | 1.103 | **0.011** | -4.174 | 0.957 | **<0.001** | -2.990 | 1.114 | **0.007** |
| European | 0.147 | 0.426 | 0.731 | -0.761 | 0.482 | 0.114 | -0.269 | 0.586 | 0.646 |
| African | 1.360 | 1.195 | 0.255 | -1.953 | 1.539 | 0.204 | -3.746 | 2.000 | 0.061 |
| Americas | 1.111 | 1.076 | 0.302 | -1.488 | 1.267 | 0.24 | -0.457 | 1.711 | 0.789 |
| Oceania | -3.034 | 1.0639 | **0.004** | -3.466 | 1.359 | **0.011** | 0.469 | 1.450 | 0.746 |
| **SEP quintile** |  |  |  |  |  |  |  |  |  |
| 1 (most disadvantaged) | 0.304 | 0.355 | 0.391 | -3.236 | 0.345 | **<0.001** | -4.459 | 0.425 | **<0.001** |
| 2 | 0.941 | 0.315 | **0.003** | -1.966 | 0.307 | **<0.001** | -2.627 | 0.397 | **<0.001** |
| 3 | 0.814 | 0.306 | **0.008** | -0.905 | 0.298 | **0.002** | -1.204 | 0.374 | **0.001** |
| 4 | 0.224 | 0.299 | 0.454 | -0.611 | 0.265 | **0.021** | -0.715 | 0.334 | **0.032** |
| 5 | referent | - | - | referent | - | - | referent | - | - |
| **Weight status** |  |  |  |  |  |  |  |  |  |
| Healthy | referent | - | - |  | - | - | referent | - | - |
| Overweight | 0.444 | 0.218 | 0.042 | -0.786 | 0.222 | **<0.001** | -2.007 | 0.298 | **<0.001** |
| Obesity | -0.721 | 0.373 | 0.053 | -3.982 | 0.383 | **<0.001** | -5.995 | 0.480 | **<0.001** |

Shaded cells indicate the MCID for Total scale score is exceeded

**Supplementary Table 4 GEE models for the association between** **PedsQL Physical summary score and Cultural and linguistic group, stratified by child age, and with adjustment for sex, SEP and weight status.**

| **Characteristic** | **Under 6**  **N=9099** | | | **6-11 years**  **N=8310** | | | **12-17 years**  **N=6791** | | |
| --- | --- | --- | --- | --- | --- | --- | --- | --- | --- |
|  | **β Coefficient** | **se** | **p** | **β Coefficient** | **se** | **p** | **β Coefficient** | **se** | **p** |
| **Female** | 0.072 | 0.237 | 0.761 | -0.590 | 0.274 | **0.032** | -1.673 | 0.341 | **<0.001** |
| **CALD group** |  |  |  |  |  |  |  |  |  |
| English | referent | - | - | referent | - | **-** | referent | - | **-** |
| Middle East & North African | -1.957 | 1.177 | 0.096 | -8.678 | 1.182 | **<0.001** | -7.111 | 1.578 | **<0.001** |
| East Asian | -1.781 | 0.678 | **0.009** | -8.433 | 0.758 | **<0.001** | -8.644 | 0.996 | **<0.001** |
| South & Central Asian | -1.617 | 1.264 | 0.201 | -7.608 | 1.179 | **<0.001** | -7.328 | 1.403 | **<0.001** |
| European | 0.391 | 0.454 | 0.389 | -1.721 | 0.531 | **0.001** | -1.425 | 0.689 | **0.039** |
| African | 1.210 | 1.334 | 0.364 | -2.923 | 1.752 | 0.095 | -6.368 | 2.379 | **0.007** |
| Americas | 1.123 | 1.154 | 0.331 | -3.165 | 1.413 | **0.025** | -2.845 | 2.095 | 0.174 |
| Oceania | -1.564 | 1.043 | 0.134 | -5.366 | 1.570 | **0.001** | -0.428 | 1.623 | 0.792 |
| **SEP quintile** |  |  |  |  |  |  |  |  |  |
| 1 (most disadvantaged) | 0.715 | 0.387 | 0.064 | -4.031 | 0.403 | **<0.001** | -5.705 | 0.505 | **<0.001** |
| 2 | 0.970 | 0.339 | **0.004** | -2.310 | 0.371 | **<0.001** | -3.696 | 0.476 | **<0.001** |
| 3 | 0.812 | 0.333 | **0.015** | -1.537 | 0.356 | **<0.001** | -1.870 | 0.445 | **<0.001** |
| 4 | 0.158 | 0.329 | 0.632 | -0.837 | 0.322 | **<0.001** | -1.077 | 0.407 | **0.008** |
| 5 | referent | - | - | referent | - | **-** | referent | - | **-** |
| **Weight status** |  |  |  |  |  |  |  |  |  |
| Healthy | referent | - | - |  | - | **-** | referent | - | **-** |
| Overweight | 0.483 | 0.241 | **0.045** | -1.062 | 0.279 | **<0.001** | -2.265 | 0.371 | **<0.001** |
| Obesity | -0.696 | 0.409 | 0.089 | -5.234 | 0.468 | **<0.001** | -7.657 | 0.597 | **<0.001** |

Shaded cells indicate the MCID for Physical Health score is exceeded

**Supplementary Table 5 GEE models for the association between** **PedsQL Psychosocial Summary Score and Cultural and linguistic group, stratified by child age, and with adjustment for sex, SEP and weight status.**

| **Characteristic** | **Under 6**  **N=9099** | | | **6-11 years**  **N=8310** | | | **12-17 years**  **N=6791** | | |
| --- | --- | --- | --- | --- | --- | --- | --- | --- | --- |
|  | **β Coefficient** | **se** | **p** | **β Coefficient** | **se** | **p** | **β Coefficient** | **se** | **p** |
| **Female** | 0.740 | 0.257 | **0.004** | 0.279 | 0.275 | 0.309 | -2.609 | 0.332 | **<0.001** |
| **CALD group** |  |  |  |  |  |  |  |  |  |
| English | referent | - | - | referent | - | - | referent | - | - |
| Middle East & North African | -0.726 | 1.017 | 0.475 | -3.620 | 1.004 | **<0.001** | 0.040 | 1.294 | 0.975 |
| East Asian | -1.979 | 0.635 | **0.002** | -2.516 | 0.604 | **<0.001** | -1.786 | 0.726 | **0.014** |
| South & Central Asian | -3.671 | 1.139 | **0.001** | -1.442 | 0.948 | 0.128 | 0.414 | 1.082 | 0.702 |
| European | -0.056 | 0.513 | 0.913 | -0.004 | 0.543 | 0.993 | 0.615 | 0.643 | 0.339 |
| African | 0.522 | 1.450 | 0.719 | -1.168 | 1.631 | 0.474 | -1.650 | 2.100 | 0.432 |
| Americas | 0.862 | 1.252 | 0.491 | -0.038 | 1.453 | 0.979 | 1.427 | 1.745 | 0.413 |
| Oceania | -4.054 | 1.236 | **0.001** | -1.796 | 1.360 | 0.187 | 1.260 | 1.521 | 0.407 |
| **SEP quintile** |  |  |  |  |  |  |  |  |  |
| 1 (most disadvantaged) | -0.059 | 0.409 | 0.886 | -2.722 | 0.376 | **<0.001** | -3.732 | 0.460 | **<0.001** |
| 2 | 0.874 | 0.373 | **0.019** | -1.661 | 0.336 | **<0.001** | -1.923 | 0.433 | **<0.001** |
| 3 | 0.829 | 0.355 | **0.020** | -0.450 | 0.324 | 0.165 | -0.814 | 0.410 | **0.047** |
| 4 | 0.247 | 0.351 | 0.483 | -0.419 | 0.295 | 0.155 | -0.596 | 0.372 | 0.109 |
| 5 | referent | - | - | referent | - | - | referent | - | - |
| **Weight status** |  |  |  |  |  |  |  |  |  |
| Healthy | referent | - | - |  | - | - | referent | - | - |
| Overweight | 0.480 | 0.260 | 0.065 | -0.577 | 0.235 | **0.014** | -1.773 | 0.320 | **<0.001** |
| Obesity | -0.754 | 0.429 | 0.079 | -3.177 | 0.396 | **<0.001** | -4.794 | 0.499 | **<0.001** |
